# Deciphering Antidiabetic, Anti‐Inflammatory, and Anti‐Cholinergic Bioactivities of *Withania somnifera* (L) Dunal Root Extract: An In Vitro and In Silico Investigation

**DOI:** 10.1002/cbdv.71409

**Published:** 2026-06-15

**Authors:** Lufuno Mulelu, Jacqueline N. Manjia, Kolawole A. Olofinsan, Londiwe Xulu, Motlalepula G. Matsabisa

**Affiliations:** ^1^ African Medicines Innovations and Technologies Development Department of Pharmacology Faculty of Health Sciences University of the Free State Bloemfontein South Africa; ^2^ Laboratory of Pharmacology and Toxicology Department of Biochemistry Faculty of Science University of Yaoundé Yaoundé Cameroon

**Keywords:** antidiabetic, anti‐inflammatory, in silico, neuroblastoma cells, protein denaturation, *Withania somnifera*

## Abstract

Alzheimer's disease (AD) is a disease associated with loss of brain cholinergic functions and a common cause of dementia. Diabetes and inflammation have been linked to some pathways contributing to AD pathogenesis. In this study, the effects of *Withania somnifera* (*W. somnifera*) root ethanol extract (WSEE) on lipoxygenase, butyrylcholinesterase (BChE), protein denaturation, and carbohydrate digestive enzymes were determined using in vitro and in silico experimental models. WSEE cytotoxicity on neuroblastoma cell lines (SH‐SY5Y) and the molecular docking simulation of its compounds were also investigated. WSEE analysis showed the presence of terpenoids, flavonoids, alkaloids, and tannins. The extract showed significant (*p* < 0.05) inhibition of protein denaturation (IC_50_ = 558.6 µg/mL), lipoxygenase (IC_50_ = 175.6 µg/mL) and BChE (IC_50_ = 313.6 µg/mL) than ibuprofen (IC_50_ = 191.1 µg/mL), vitamin C (IC_50_ = 44.97 µg/mL), and rivastigmine (IC_50_ = 123.7 µg/mL), respectively. It also inhibited α‐glucosidase (IC_50_ = 343.0 µg/mL) and reduced SH‐SY5Y cell viability at ≥ 80 µg/mL. The docking studies revealed that sitoindoside IX, somniferin, withanolide B, and withasomniferolide B in the plant exhibit high binding affinities, where withasomniferolide B presents the highest docking scores (−11.4 kcal/mol) with α‐amylase. Further in vivo and molecular studies are needed to validate the plant's usefulness in AD management.

## Graphical abstract

In this investigation, *Withania somnifera* root ethanol extract displays anti‐inflammatory and antidiabetic properties. Though compounds including withasomniferolide B and sitoindoside IX have been previously identified as phytoconstituents in the plant, in silico analysis from this study revealed them as new lead compounds that could act as potential inhibitors of α‐amylase and lipoxygenase.

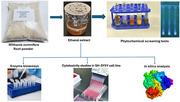

## 1 Introduction

Neurodegenerative diseases (NDDs) encompass a range of disorders that primarily damage neurons in the human brain, leading to progressive impairments in mobility and cognitive functions [1]. Among the various NDDs, Parkinson's disease, Huntington's disease, amyotrophic lateral sclerosis, and Alzheimer's disease (AD), which is the most prevalent form of dementia, currently affects over 50 million people worldwide [2]. The aetiology of these diseases is multifactorial, involving complex interactions among age, environmental factors, lifestyle choices, and genetic predispositions. In low‐ to middle‐income countries, such as South Africa, approximately 4.4% of individuals over 60 years are affected by dementia, with projections indicating a rise to 7 million cases by 2030 in Bloemfontein alone [3].

Despite significant research efforts aimed at understanding the pathways involved in AD, such as oxidative stress and neuroinflammation, the accumulation of amyloid‐beta (Aβ) plaques, and neurofibrillary tangles (NFTs) remains the primary pathological hallmark of the disease [4]. The progression of AD is characterized by a gradual decline in acetylcholine, a neurotransmitter critical for learning and memory, due to its hydrolytic degradation by cholinesterase enzymes, specifically acetylcholinesterase (AChE) and butyrylcholinesterase (BChE) [5]. Notably, studies have shown that BChE levels increase more significantly than AChE levels in the brains of AD patients, exacerbating acetylcholine degradation [6]. The resulting decline in cholinergic signaling contributes to neuronal death and cognitive decline, manifesting in clinical symptoms such as personality changes, emotional difficulties, and behavioral abnormalities [7, 8].

Furthermore, inflammation and type 2 diabetes have been implicated in the pathogenesis of many diseases, including AD [9]. The activity of lipoxygenase, an enzyme that converts arachidonic acid to leukotrienes, has been implicated in inflammation and neurotoxicity in the central nervous system [10]. Besides this mechanism, oxidative stress mediated by the formation of advanced glycation end products (AGEs) contributes to inflammation, particularly in the brain [11]. Additionally, the dysregulation of alpha‐amylase and alpha‐glucosidase enzymes further disrupts glucose homeostasis, thereby exacerbating insulin resistance in type 2 diabetes. This impairment in glucose metabolism can adversely affect brain function, leading to cognitive decline and increasing the risk of developing AD. Thus, the interplay between insulin resistance and the activities of alpha‐amylase and alpha‐glucosidase underscores the significance of metabolic health in the pathophysiology of neurodegenerative disorders, including AD [9, 12]

Currently, there is no cure for AD, and treatment strategies primarily focus on managing symptoms and slowing disease progression [13]. Cholinesterase inhibitors such as donepezil, rivastigmine, and galantamine are commonly prescribed, but they are associated with adverse effects, which include gastrointestinal distress and cardiovascular issues [14]. As a result, there is a growing need for novel therapeutic approaches to minimize side effects. Recent pharmacological research has turned to medicinal plants, which appear to offer a promising avenue for discovering new, better‐acting treatments with fewer adverse effects [15].

Several plant extracts have shown potential in treating AD. For instance, *Valeriana carnosa* has demonstrated AChE inhibitory effects and alleviated anxiety and sleep disturbance [16]. Curcumin is known for its antioxidant and anti‐inflammatory properties, which improve cognitive function in patients with AD. Additionally, extracts from *M. alysson* and *P. harmala* have been reported to inhibit BChE activity while displaying significant anti‐inflammatory effects [17].

*Withania somnifera*, popularly known as Ashwagandha, is a member of the Solanaceae family and has been widely utilized in traditional medicine to treat various health conditions, including diabetes and cognitive dysfunction [13]. The crude extracts and isolated compounds, including the steroidal lactones (withaferin A and withanolides) of this plant, exhibit notable anti‐inflammatory, antioxidant, and immunomodulatory properties [18]. *Withania somnifera* is rich in phytochemical constituents, particularly bioactive compounds from the steroidal lactone class. Among these, withaferin A has been identified as having potent anti‐inflammatory and antidiabetic properties. It works by targeting NF‐κB pathways, reducing levels of inflammatory cytokines (such as IL‐6 and TNF‐α), and improving glucose uptake in muscle/fat cells. Additionally, Withanolide A and B possess neuroprotective effects and have been studied for their anti‐Alzheimer's bioactivity since they inhibit AChE, a key enzyme involved in AD. Furthermore, these compounds promote neurite outgrowth, indicating their potential in cognitive health [19]. Recent research has identified the ethanolic extracts of *W. somnifera* as effective inhibitors of AChE, demonstrating potential to reduce neuronal dysfunction and Aβ protein accumulation [20]. However, studies examining the effectiveness of *W. somnifera* against BChE and its role in managing type 2 diabetes remain limited, highlighting a significant gap in the pursuit of effective anti‐Alzheimer's therapeutics. This study investigated the anti‐inflammatory, cholinesterase‐inhibitory, and antidiabetic properties of *W. somnifera* with the aim of evaluating its potential as a therapeutic option for mitigating complications could increase AD progression.

## 2 Materials and Methods

### 2.1 Plant Collection and Extraction

*W. somnifera* root powder was purchased at Essentially Natural Supermarket in Cape Town, South Africa, and a sample of the botanical material, tagged as a specimen (FF00013), was kept in the plant storage of African Medicines Innovations and Technologies Development (AMITD) unit, University of the Free State. 200g of the dry root powder was weighed and macerated in 2 L of ethanol for 72 h on a shaker set at 150 rpm [21]. After 72 h, the extract was filtered using Whatman filter paper. This step was followed by sample concentration in a rotary evaporator with a 30°C water bath, 120 mbar vacuum, and a Chiller set to 5°C. The recovered sample was transferred to a glass plate and air‐dried in the fume hood before the percentage yield was determined to be 0.6%.

### 2.2 Qualitative Phytochemical Screening of WSEE

Several classes of phytochemicals have been detected in *W. somnifera* extracts. Specific chemical reagents were prepared to identify specific groups of phytochemicals in the root. The tests were conducted using the modified method of Arya and Chauhan [22].

#### 2.2.1 Test for Terpenoids

Approximately 5 mg of extract was dissolved in a 5 mL solution composed of 2 mL of chloroform and 3 mL of concentrated sulfuric acid, resulting in a reddish‐brown coloration interface that was expected to form, indicating positive results for the presence of terpenoids.

#### 2.2.2 Test for Flavonoids

Approximately 5 mg of extract was dissolved in ethanol, and then a piece of magnesium chip and 5,6 drops of HCl were added, with the expectation of an immediate development of a magenta color, which indicates the presence of flavonoids.

#### 2.2.3 Test for Alkaloids

To 1 mL of Dragendorff's reagent, 2 mL of the ethanolic extract was added, whereby an orange‐red precipitate was expected to form, indicating the presence of alkaloids.

#### 2.2.4 Test for Tannins

About 5 mg of extract, macerated in 2 mL of ethanol, was mixed with 1 mL of aqueous potassium dichromate solution. A color change to a yellow‐brown color solution indicates the presence of tannins.

#### 2.2.5 Test for Phenols

The extract was mixed with 1 mL of water, followed by the addition of one or two drops of 10% FeCl_3_. A blue, green, purple, or red color indicates the presence of phenols.

Depending on the expected color changes that depict the presence of each phytochemical class, the outcome of each test was observed visually, and inferences were determined as present (+) when there is a light color change, Absent (‐) when there is no color change and Highly present (+++) when there is a deep color change.

### 2.3 Thin‐Layer Chromatography (TLC) Profile of WSEE

TLC was performed for qualitative analysis of possible compounds in the root extract, using a modified method described by Meena et al. [23]. This was done to establish a phytochemical fingerprint for the identification of *W. somnifera*. A silica gel (60F_254_) TLC plate (Merck, Germany) was used for the TLC. An elution solvent of ethyl acetate and diethyl ether (9:1) was prepared and constituted the eluting or developing solvent system. The WSEE was dissolved in ethanol (0.5 mg/mL), and 10 µL of dissolved extract was manually applied to the plate as a spot using a microcapillary pipette, positioned 1 cm from the bottom and 1.5 cm from the side of the plate. The spotted TLC plate was air‐dried, placed in a TLC tank containing 40 mL of the eluting solvent system, and covered with a glass lid to saturate the tank with the mobile phase vapor. The developed plate was removed from the chamber and allowed to air‐dry. Visualization of the developed spots was performed under 366 nm UV light.

### 2.4 Butyrylcholinesterase Inhibitory Effects of WSEE

A modified version of the Ellman colorimetric method was used to determine the BChE inhibition assay [24]. Three buffers prepared were used for the assay: Buffer A (50 mM Tris‐HCl, pH 8), Buffer B (50 mM, pH 8, containing 0.1% bovine serum albumin), and Buffer C (50 mM Tris‐HCl, pH 8, containing 0.1 M NaCl, and MgCl_2_.6H_2_O (0.02 M). In this method, 50 µL of different concentrations of WSEE in DMSO (640, 320, 160, 80, 40, and 20 µg/mL) were mixed in a cuvette with 50 µL of 15 mM of butyrylcholine chloride in distilled deionized, 250 µL of 3 mM DTNB in Buffer C, and 400 µL of Buffer B. absorbance of this mixture was then measured thrice for each concentration at 405 nm every 15 s.

Subsequently, 50 µL of 1.5 µg/mL BChE (Human, Sigma–Aldrich, Cat no: B4186) was added to the cuvette, and the absorbance was measured for 5 mins, with readings taken every 15 s. Rivastigmine (640, 320, 160, 80, 40, and 20 µg/mL) was used as a positive control. Any absorbance increase caused by spontaneous substrate hydrolysis was adjusted by subtracting the absorbance before adding the enzyme from the absorbance after the enzyme was added. The inhibition percentages were derived from the enzyme reactions using the following formula.

$$\mathrm{Inhibition}\left(\%\right)=\left[\left(A-B\right)/A\right]\times 100$$

where A is the absorbance without the test sample and B is the change in absorbance in the presence of the test sample.

### 2.5 Anti‐Inflammatory Activities of WSEE

#### 2.5.1 Inhibition of Protein Denaturation

The reaction mixture (3 mL) consisted of 50 µL of extract or ibuprofen at various concentrations (20, 40, 80, 160, 320, and 640 µg/mL) and 0.45 mL of egg albumin (3% aqueous solution), along with 2500 µL of phosphate‐buffered saline (pH 6.3; 0.1 M). Distilled water was used as a negative control, without egg albumin. Thereafter, the mixtures were incubated for 15 min at 37°C, followed by heating for 5 min at 70°C. The reaction mixture was allowed to cool to ambient room temperature for 15 min. Turbidity was measured spectrophotometrically at 660 nm. The protein denaturation percentage inhibition was calculated using the formula according to Kumari et al. [25].

$$\begin{array}{ccc} & & \mathrm{Inhibition}\;\mathrm{Percentage}\\ & & \;=\frac{\mathrm{Abs}\;\mathrm{Control}-\mathrm{Abs}\;\mathrm{treated}}{\mathrm{Abs}\;\mathrm{Control}}\;\times \;100 \end{array}$$

#### 2.5.2 Inhibition Effect on Lipoxygenase Enzyme Activity

This assay was performed according to the procedure of Manjia et al. [26] with slight modifications. 200 U/mL 15‐lipoxygenase (*Glycine max*, Sigma‐Aldrich, Cat no: L7395) was incubated for 5 min with 30 µL of extract or ascorbic acid (640, 320, 160, 80, 40, and 20 µg/mL) at room temperature. The mixture was then incubated at room temperature for an additional 20 min in the dark after the addition of 40 µL of linoleic acid (140 µM). 100 µL of the FOX reagent, which is composed of sulfuric acid (30 mM), xylenol orange (100 µM), iron (II) sulphate (100 µM), and a methanol/water mixture (9:1), was added to terminate the enzyme reaction. After 20 min in the dark, the absorbance values were measured at 560 nm to determine lipoxygenase inhibitory activity using the formula below

$$\begin{array}{ccc} & & \mathrm{LOX}\;\mathrm{Inhibition}\;\left(\%\right)\\ & & \;=\left[\frac{\mathrm{Absorbance}\;\mathrm{of}\;\mathrm{control}\;-\mathrm{Absorbance}\;\mathrm{of}\;\mathrm{sample}}{\mathrm{Absorbance}\;\mathrm{of}\;\mathrm{control}}\right]\;\times 100 \end{array}$$

### 2.6 Anti‐Diabetic Activities of WSEE

#### 2.6.1 Inhibitory Effect on α‐Amylase Enzyme

With minor adjustments, a prior method of Mustafa et al. [27] was used to determine alpha‐amylase (Porcine pancreas, Sigma–Aldrich, Cat no: A6255) activity of WSEE. The samples were dissolved in 0.02 M sodium phosphate buffer (pH 6.9) made to final concentrations ranging from 20 to 640 µg/mL. Before incubation for 10 min at 37°C, the dissolved samples (100 µL) were mixed with 0.5 mg/mL α‐amylase solution (100 µL) in 0.02 M sodium phosphate buffer (pH 6.9). A 100 µL of a 1% starch solution (in 0.02 M sodium phosphate buffer, pH 6.9) was prepared and added to the solution, which was then incubated for an additional 30 min at 37°C. The mixture was further supplemented with 200 µL of DNS (3,5‐dinitrosalicylic) color reagent. To stop the reaction, the test tubes were incubated for 5 min in a water bath set at 100°C. Following incubation, 40 µL of the reaction mixture was added to 210 µL of water, and the absorbance was measured at 550 nm. Acarbose was used as a positive control for the experiment. The following formula was used to determine the % α‐amylase inhibition:

$$\mathrm{Inhibition}\;\left(\%\right)=\frac{\mathrm{Abs}\;\mathrm{Control}-\mathrm{Abs}\;\mathrm{treated}}{\mathrm{Abs}\;\mathrm{Control}}\;\times 100$$

### 2.7 Inhibitory Effect on α‐Glucosidase Enzyme

The α‐glucosidase inhibition was determined by the following modified methods of Mechchate et al. [28] and Olofinsan et al. [29]. A 96‐well plate containing 100 µL of alpha‐glucosidase (*Saccharomyces cerevisiae*, Sigma–Aldrich, Cat no: G5003) was supplemented with 100 µL of each plant extract at varying concentrations, and acarbose was used similarly as the positive control. The plate was incubated for 10 min at 37°C. After incubation, 30 µL of the pNPG (4‐nitrophenyl‐β‐D‐glucopyranoside) substrate was added, and the absorbance was measured at 405 nm 1 min after the addition of pNPG. The following formula was used to determine the % α‐glucosidase inhibition.

$$\mathrm{Inhibition}\;\left(\%\right)=\frac{\mathrm{Abs}\;\mathrm{Control}-\mathrm{Abs}\;\mathrm{treated}}{\mathrm{Abs}\;\mathrm{Control}}\;\times 100$$

### 2.8 Cytotoxicity of WSEE in Neuroblastoma SH‐SY5Y Cells

#### 2.8.1 Maintenance of Cell Culture

Complete media containing Dulbecco's Modified Eagle's Medium F12 (DMEM‐F12) with high glucose concentration of 45 g/L D‐glucose, L‐glutamine, and 25 mM HEPES (Gibco, Life Technologies), supplemented with 10% foetal bovine serum (FBS), were used to culture ATCC SH‐SY5Y cells (RRID: CVCL_0019) at 37°C in a humidified incubator with 5% CO_2_. When the cells reached 70%–80% confluence, they were trypsinized using 0.25% (w/v) trypsin‐EDTA (Gibco, Life Technologies) and rinsed twice with 5 mL of PBS (pH 7.2). For additional passaging, the cells were sub‐cultured every 3 to 4 days at a sub‐cultivation ratio of 1:2 to 1:4. The cell viability was checked with trypan blue 0.4% (Cytiva Hyclone, Logan, UT, USA) using the Countess automated cell counter (Thermofisher Scientific), and only cell suspensions with a cell viability greater than 90% were used for subsequent cell‐based assays, following the methods of Siddiqui et al. [30].

#### 2.8.2 Cytotoxicity Screening in SH‐SY5Y Cells

On a 96‐well plate (Nunc, Thermofisher Scientific), SH‐SY5Y cells were seeded at a density of 10 000 cells/well [31]. Each well received 100 µL of medium, and the cells were left to adhere overnight at 37°C with 5% CO_2_. Following attachment, the media was aspirated, and cells were exposed to different concentrations of WSEE (20, 40, 80, 160, 320, and 640 µg/mL) for 48 h. Doxorubicin (3 µg/mL) was employed as a positive control. After treatment, the used media was aspirated. Then, 100 µL of fresh medium containing 2‐(2,5‐dimethyl‐2‐thiazolyl)‐2,5‐diphenyl‐2H‐tetrazolium bromide (MTT) was added, and the mixture was incubated for 2°h at 37°C. Following incubation, the MTT solution was removed, and 100 µL of DMSO was added [32]. The plates were gently shaken for 5 min. Absorbance was measured at 550 nm using a Thermo Scientific Multiskan GO spectrophotometer. The percentage of cell viability was calculated using the formula below:

$$\mathrm{Cell}\;\mathrm{Viability}\left(\%\right)=\left[\left(A_{0}-A_{1}\right)/\left(A_{2}-A_{1}\right)\right]\times 100$$

*A*_0_ is the absorbance of the sample; *A*_1_ is the absorbance of the blank, and *A*_2_ is the absorbance of the negative control (0.5% DMSO).

#### 2.8.3 Molecular Docking of *W. somnifera*‐Derived Compounds

A literature search was conducted to identify the major chemical compounds present in *W. somnifera* root extracts. An in‐house library of 17 compounds reported in the root was determined from previous reports [33, 34, 35], and then used for molecular docking studies. The 3D structures of all these phytochemicals were retrieved in SDF format from the PubChem online website and individually imported to the working directory of the Autodock Vina Software. After polar hydrogen molecules and Kollman charges were added to the ligand structures, the resulting structures were saved in a pdbqt file format. Similar three‐dimensional structures of metabolic proteins, including α‐amylase [36] (PDB ID: 1B2Y), BChE [37] (PDB ID: 6ESJ), α‐glucosidase [38] (PDB ID: 3L4W), and lipoxygenase [39] (PDB ID: 1N8Q), with resolutions of 3.20, 2.98, 2.00, and 2.10 Å, respectively, were also downloaded from the online Protein Data Bank website. The nonstandard atoms and the co‐crystallized water molecules attached to protein structures were initially removed with Discovery Studio software before the pdb file output of the program was subject to a similar procedure employed for the ligands. Molecular docking of each compound was performed with its respective protein using the gradient‐based local search genetic algorithm of AutoDock Vina, as described previously [40, 41]. Using 1Å default grid spacing, the docking grid box *X*, *Y*, *Z* center coordinate was set at 16:13.19:17 for α‐amylase, 30:30:30 for BChE, 30:30:0 for α‐glucosidase, and 33:33:33 for lipoxygenase. The molecular interactions of the best‐binding poses of each compound with the respective proteins were visualized in 2D and 3D using the previously mentioned Discovery Studio software [42].

#### 2.8.4 Statistical Analysis

Technical triplicate assays were done (*n* = 3) for each sample, and the outcomes were presented as mean ± SD. Data analysis was performed using GraphPad Prism V8.0.2. Statistically significant differences between the extract and the standard compound experimental groups were established using *t*‐tests at *p* < 0.05.

## 3 Results

### 3.1 WSEE Phytochemical Analysis and Profiling

**FIGURE 1:**
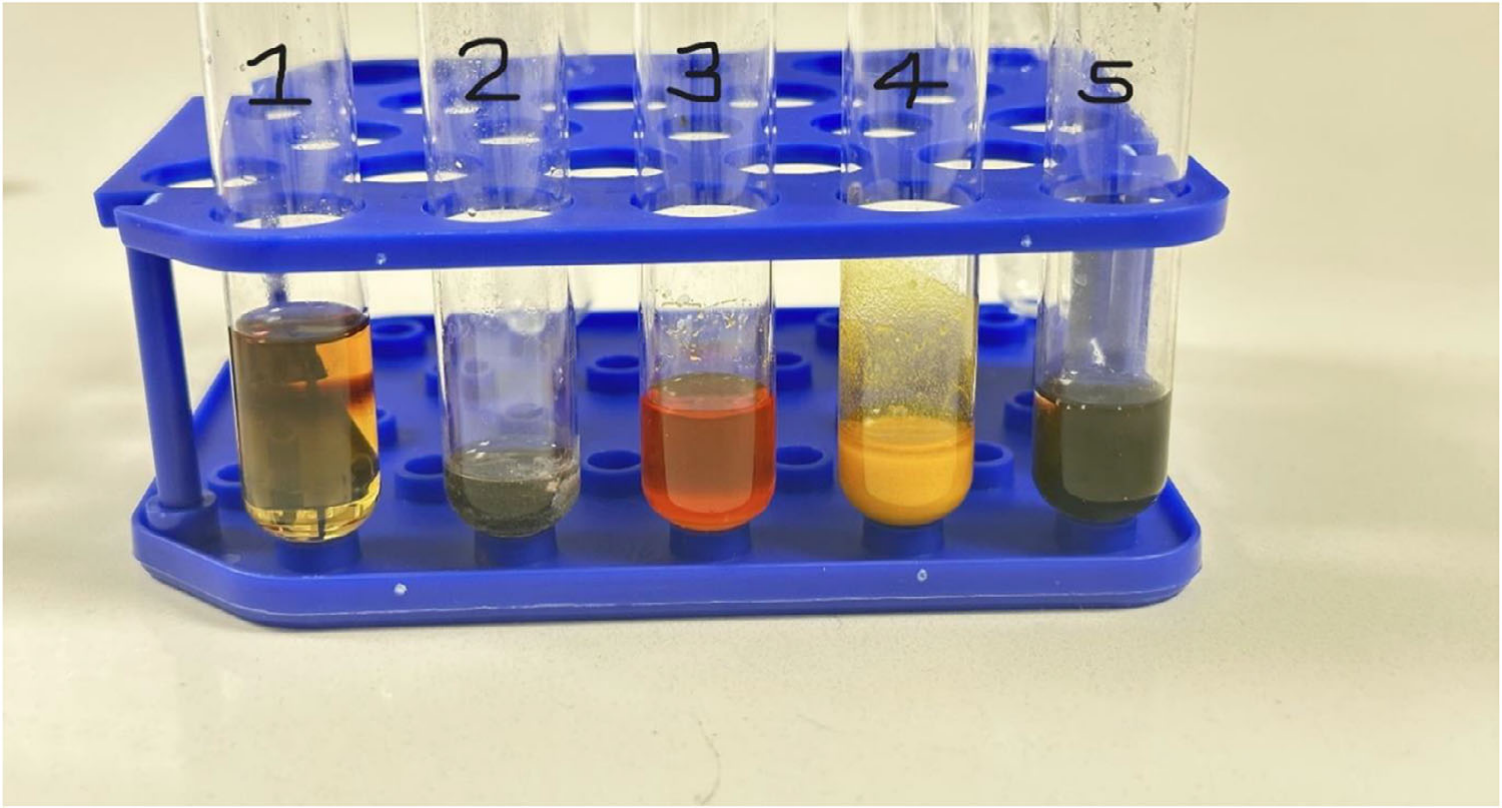
Phytochemical screening tests of *W. somnifera* root extract for (1) Terpenoids, (2) Flavonoids, (3) Alkaloids, (4) Tannins, and (5) Phenols.

**FIGURE 2:**
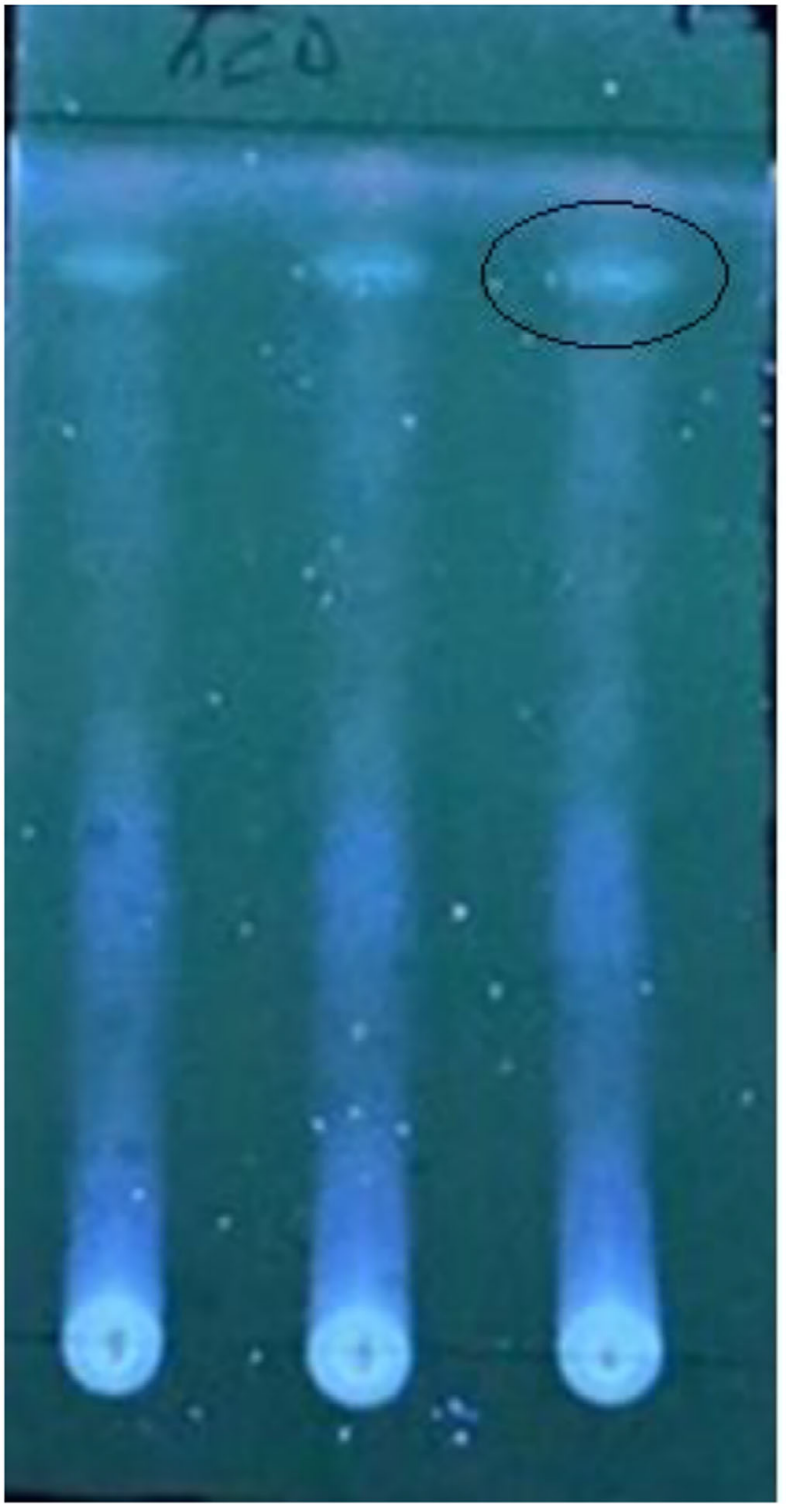
The TLC plate chromatogram of the *W. somnifera* root extract spotted in triplicate and viewed under UV light at 366 nm.

Thin‐layer chromatography is often used to visualize the profiles of the various components in a plant extract. Before running the separation analysis, the results of the phytochemical test for the different classes of phytoconstituents in the WSEE root (Figure 1) and presented in Table 1 revealed the presence of flavonoids, terpenoids, alkaloids, and tannins. Moreover, the TLC phytochemical profiling of the plant extract using the ethyl acetate/diethyl ether solvent system, as presented in Figure 2, revealed a prominent spot (circled) with a retention factor (*R*_f_) of 0.56. Although no standard compound was spotted along with the extract sample, this spot could represent one of the biologically active components of the *W. somnifera* root.

**TABLE 1:** Qualitative inference for *W. somnifera* root extract phytochemical screening result.

	Terpenoids	Flavonoids	Alkaloids	Tannins	Phenols
Inferences	+	+	+	+++	—

+ = Present;—= Absent; +++ = Highly present.

### 3.2 WSEE Effect Butyrylcholinesterase Activity

**FIGURE 3:**
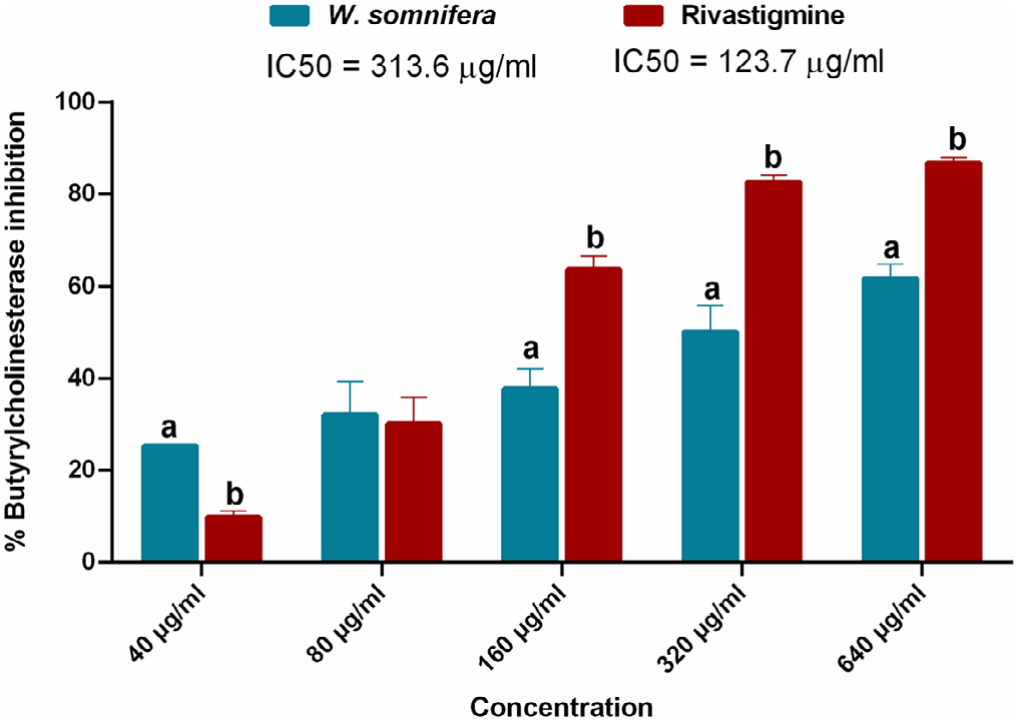
*W. somnifera* root extract inhibitory effect butyrylcholinesterase enzyme. Data were presented as Mean ± SD, with *n* = 3 representing the number of experiments. Different letters (a or b) on bars at each concentration denote statistically significant differences in the tested specimen (p < 0.05).

In Figure 3, BChE enzyme inhibition increases with increasing concentration of the root extract. At 40 µg/mL, the inhibitory effect of the plant extract was approximately 61% higher than that of rivastigmine. Despite its better efficacy at the low concentration and comparable effect at 80 µg/mL, the extract, with an IC_50_ of 313.6 µg/mL, did not perform to the levels of the standard drug (IC_50_ = 123.7 µg/mL) at higher test concentrations. At 320 and 640 µg/mL, the inhibitory effects of the extract on the enzyme were about 40% and 29% lower than those of the chemical compound.

### 3.3 WSEE Effect on Protein Denaturation and Lipoxygenase Activity

**FIGURE 4:**
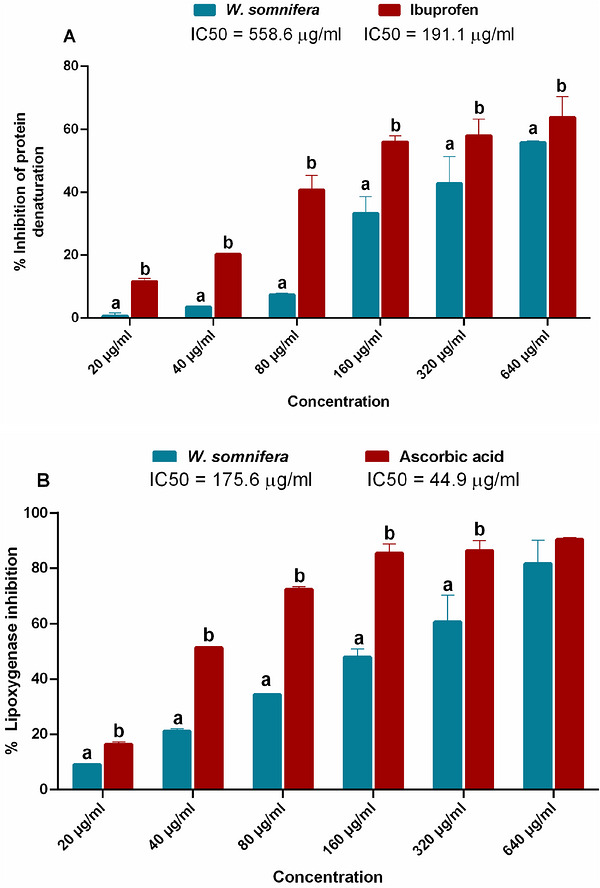
*W. somnifera* root extract inhibitory effects on (A) Protein denaturation and (B) Lipoxygenase enzyme activity. Data were presented as Mean ± SD, with *n* = 3 representing the number of experiments. Different letters (a or b) on bars at each concentration denote statistically significant differences in the tested specimen (p < 0.05).

At all the test concentrations in Figure 4A, *W. somnifera* root extract showed lower protein denaturation inhibition than ibuprofen. The extract showed approximately 82%, 40%, 26%, and 13% lower inhibition than the anti‐inflammatory drug at 80 to 640 µg/mL concentrations. Moreover, a similar pattern was observed in Figure 4B, where, even at 20–40 µg/mL lower experimental concentrations, the inhibitory effect of ascorbic acid on lipoxygenase was about 82% and 59% higher than that of the plant extract. Overall, the extract's IC_50_ for the enzyme was 175.6 µg/mL, compared with 44.9 µg/mL for ascorbic acid.

### 3.4 WSEE Effect on Carbohydrate Digestive Enzyme Activities

**FIGURE 5:**
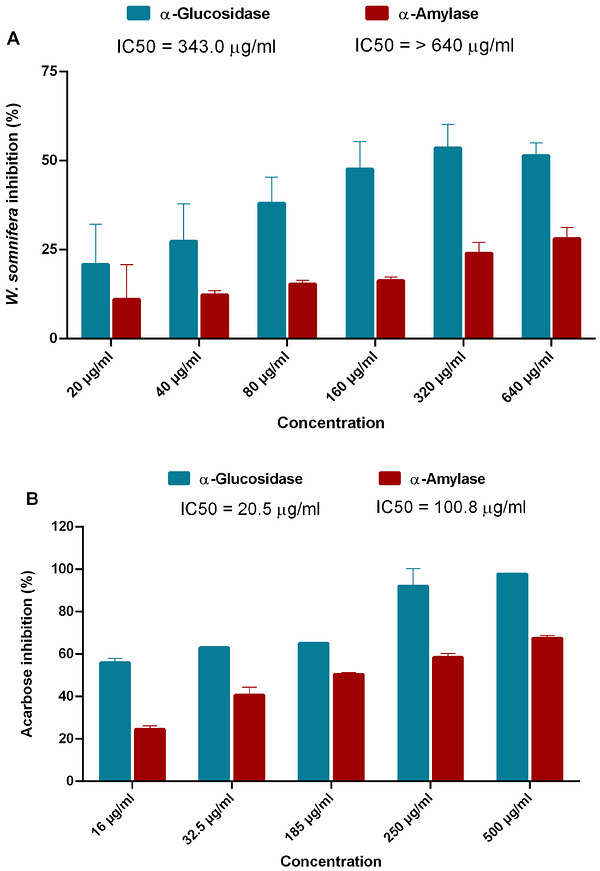
*W. somnifera* root extract inhibitory effect on carbohydrate digestive enzymes compared with acarbose standard. Data were presented as Mean ± SD, with *n* = 3 representing the number of experiments.

Figure 5 compares the inhibitory effect of the studied plant extract on carbohydrate digestive enzymes with the similar activity of acarbose, a standard antidiabetic drug. Although the plant exhibited α‐glucosidase inhibitory activity, the extent of inhibition was lower with α‐amylase (Figure 5A).  At the plant test concentrations, the IC_50_ of the extract for α‐glucosidase was 343 µg/mL, which was 73% higher than for α‐amylase. Contrastingly, in Figure 5B, the lower IC_50_ values of acarbose against the extract compared to the digestives (α‐amylase = 100.8 µg/mL; α‐glucosidase = 20.5 µg/mL) suggest a lower efficacy of the latter over the former.

### 3.5 WSEE Effect on SH‐SY5Y Cell Viability

**FIGURE 6:**
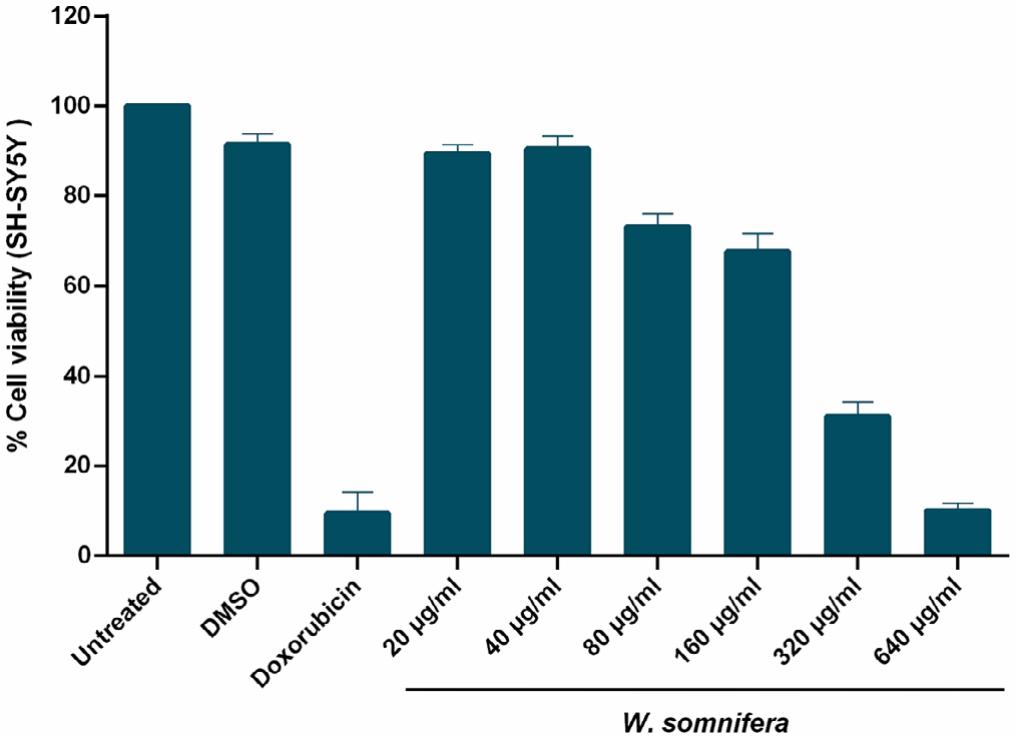
SH‐SY5Y neuroblastoma cell line cell viability after treatment with various concentrations of *W. somnifera* root extract. Data were presented as Mean ± SD, with *n* = 3 representing the number of experiments.

**FIGURE 7:**
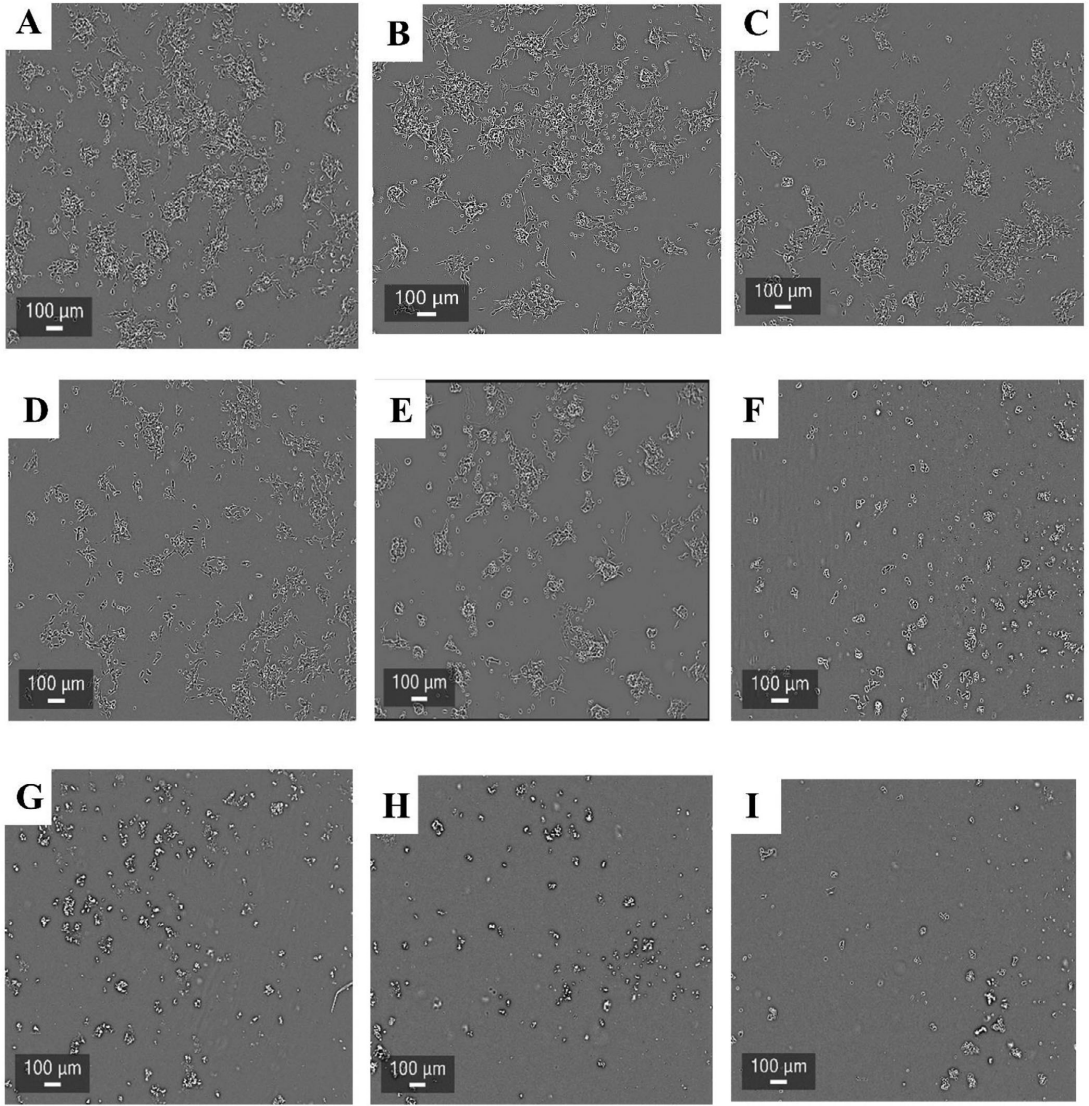
Morphological change in SH‐SY5Y cell line after treatment with culture media containing (A) DMEM‐F12 only, (B) DMEM‐F12 and 0.64% DMSO, (C) DMEM‐F12 and 20 µg/mL *W. somnifera* extract, (D) DMEM‐F12 and 40 µg/mL *W. somnifera* extract, (E) DMEM‐F12 and 80 µg/mL *W. somnifera* extract, (F) DMEM‐F12 and 160 µg/mL *W. somnifera* extract, (G) DMEM‐F12 and 320 µg/mL *W. somnifera* extract, (H) DMEM‐F12 and 640 µg/mL *W. somnifera* extract, and (I) DMEM‐F12 and 3 µg/mL Doxorubicin.

The cytotoxicity of *W. somnifera* root extract on the tested cancer cells, as presented in Figure 6, increases with increasing concentration, ranging from 80 to 640 µg/mL. Although the effect of the extract treatment on SH‐SY5Y cells was not statistically different from that of the DMSO blank at 20–40 µg/mL, the extract's toxicity at 640 µg/mL was comparable to that of the doxorubicin chemical standard. Indeed, the image data in Figure 7 revealed a significant reduction in the population of live cells at the highest test concentration (*A*) compared to the normal control experiment (*I*).

### 3.6 WSEE Phytocompounds Molecular Docking Analysis

**FIGURE 8:**
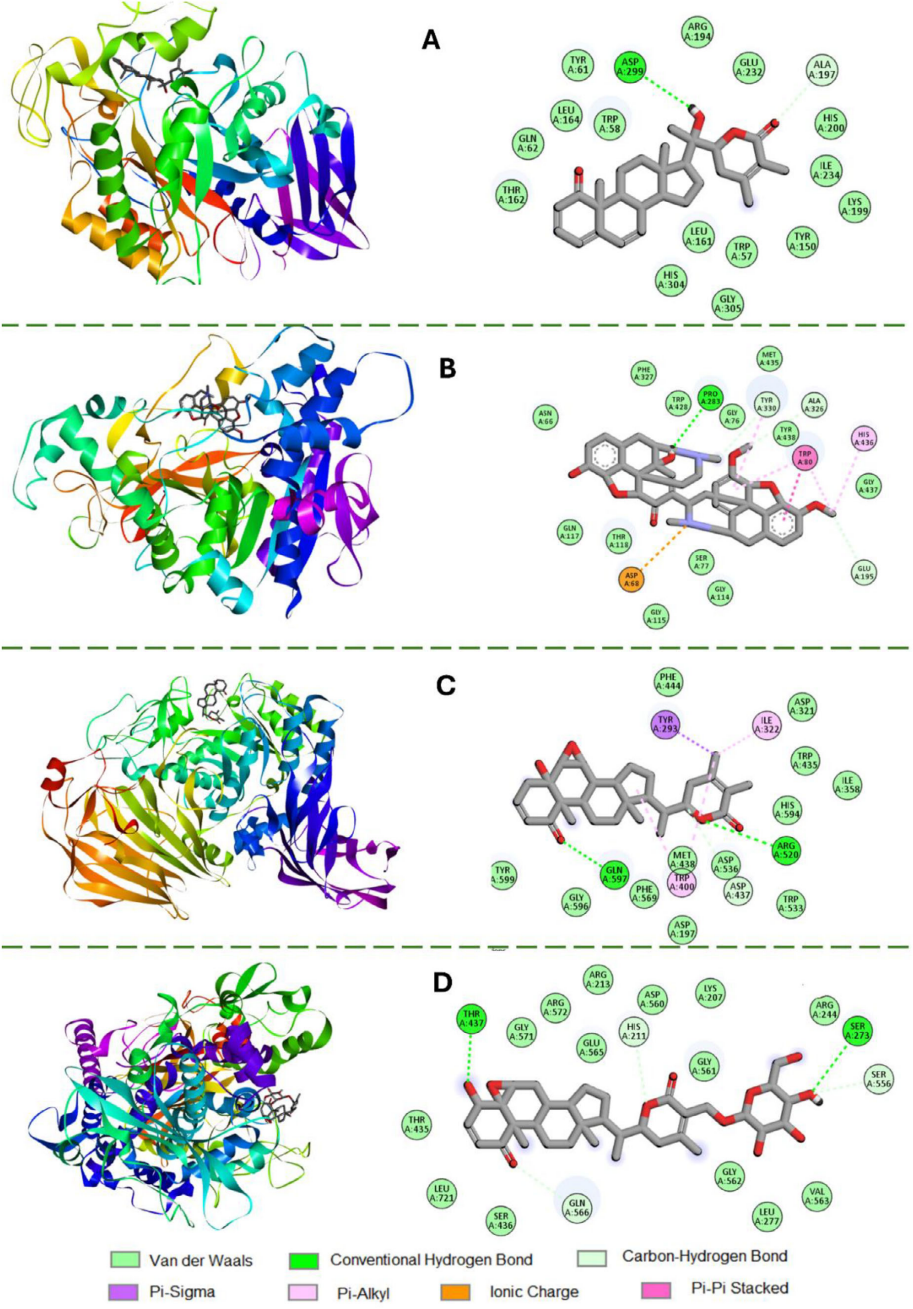
Molecular interactions in 2D and 3D of (A) Withasomniferolide B with α‐amylase, (B) Somniferin with butyrylcholinesterase, (C) Withanolide B with α‐glucosidase, and (D) Sitoindoside IX with lipoxygenase.

The calculated binding energy scores for the *W. somnifera* root compounds and the selected protein targets are presented in Table 2. Lower docking scores below −11.0 kcal/mol were displayed by withanolide B, withanolide G, withasomniferolide A, and withasomniferolide B against the α‐amylase enzyme. Somniferin had the strongest docking score with BChE (−12.2 kcal/mol), whereas sitoindoside IX produced similar affinity with lipoxygenase (−9.5 kcal/mol). While withanolide B had a calculated score of −9.9 kcal/mol with α‐glucosidase, other compounds, including withaferin A, withasomidienone, and withasomniferolide B, showed lower affinities for the carbohydrate digestive enzyme, with docking values of −9.8, −9.3, and −9.7 kcal/mol, respectively. Ascorbic acid had a score of −5.6 kcal/mol after molecular docking with lipoxygenase, while acarbose and rivastigmine, standard employed during an in vitro experiment, had a computed value of −7.3 kcal/mol with other proteins. The 2D molecular interaction image in Figure 8A revealed that withasomniferolide B integrated more strongly with ASP299 and ALA197 of α‐amylase. When considering the 2D images related to somniferin 8B, it could be observed that the compound forms ionic charges, hydrogen bonds, pi─pi stacked bonds, carbon─hydrogen bonds, and pi─alkyl bonds with various amino acid residues of BChE. In Figure 8C, withanolide B presented two stronger hydrogen bonds with GLN596 and ARG520 of BChE. Contrastingly, in Figure 8D, sitoindoside IX showed a similar interaction with THR437 and SER273 at the active site of lipoxygenase.

**TABLE 2:** Binding energies (kcal/mol) from molecular studies of *W. somnifera* root selected compounds with cholinergic, inflammatory and carbohydrate digestive enzyme targets.

Compound	α‐Amylase	Butyrylcholinesterase	α‐Glucosidase	Lipoxygenase
Anaferine	−6.4	−7.0	−6.5	−6.9
Cuscohygrine	−6.5	−6.3	−5.6	−5.9
Isopelletierine	−4.9	−5.1	−5.3	−5.3
Pseudotropin	−5.6	−5.4	−5.6	−4.4
Sitoindoside IX	−10.6	−11.7	−8.2	−9.5
Somniferin	−8.5	−12.2	−8.4	−8.5
Tropine	−5.6	−5.3	−5.6	−4.5
Withaferin A	−10.5	−11.0	−9.8	−8.0
Withanolide A	−10.6	−10.6	−8.8	−8.6
Withanolide B	−11.4	−10.4	−9.9	−8.7
Withanolide G	−11.2	−10.7	−8.9	−8.6
Withanone	−9.0	−9.8	−8.7	−8.4
Withanoside IV	−7.5	−11.7	−8.5	−8.3
Withanoside V	−7.8	−11.9	−8.5	−8.1
Withasomidienone	−10.5	−11.6	−9.3	−8.3
Withasomniferolide A	−11.1	−10.6	−8.9	−8.8
Withasomniferolide B	−11.5	−11.4	−9.7	−8.8
Acarbose	−7.3	—	−7.3	—
Rivastigmine	—	−7.3	—	—
Ascorbic acid	—	—	—	−5.6

## 4 Discussion

The use of medicinal plants for disease management has been a traditional practice for centuries. *W. somnifera*, a small shrub plant of the family Solanaceae, and sometimes regarded as Indian ginseng, has been described locally as an anti‐stress medicine [43]. The powdered, dried root samples extract of the plant (WSEE) was investigated for its potential application in modulating proteins linked to neurovegetative diseases. The results suggest that WSEE suppressed inflammatory markers associated with certain neurological disorders and has shown potential antidiabetic properties.

The pharmacological activities of plants have been linked to various classes of chemical compounds found within them. Tannins have been described to deter such plants from being consumed by herbivores [44]. Other compounds, such as terpenoids, have been reported as signaling molecules that facilitate plant‐to‐plant communication while also playing a role in growth regulation [45]. The TLC analysis using the ethyl acetate‐diethyl ether (9:1) solvent mixture showed a spot with *R*_f_ of 0.56, which could be ascribed to one of the compounds in Table 1. In the previous investigation by Meena et al. [23], withanolide A, used as a standard for the high‐performance TLC fingerprint profile of a formulation containing *W. somnifera*, was detected at an *R*_f_ of 0.45 in a toluene: ethyl acetate formic acid solvent system. While the prominent spot we observed in Figure 2 could be withanolide A, differences in *R*_f_ may be due to the different solvent system used in the present investigation. Since Table 1 reveals that WSEE has high levels of tannins, the findings from the current work are in line with Singh et al. [46], where higher tannin levels were measured in *W. somnifera* roots than in their corresponding leaf parts.

Physiological levels of the Aβ protein have been described to perform various critical functions in the brain. According to Morley et al. [47], concentrations as low as picomolar amounts of this protein have been reported to promote memory, learning, and synaptic plasticity in the hippocampus of animal models. Despite these roles, the abnormal degradation of Aβ protein by beta‐ and gamma‐secretase enzymes results in the former protein becoming denatured and subsequently aggregating around neurons, forming plaque structures implicated in the pathophysiology of AD [48]. A previous study by He, et al. [49] described the role of inflammatory markers, such as 5‐lipoxygenase, in contributing to Alzheimer's development by elevating gamma‐secretase activity. WSEE dose‐dependently inhibited protein denaturation and lipoxygenase activity (Figure 4). This result suggests a potential therapeutic efficacy that could be further explored in the management of neurodegenerative disorders. In a previous study by Nile et al. [50], the IC_50_ of *W. somnifera* root ethanol extract was 22.97 µg/mL. The differences between this result and the 175.6 µg/mL obtained in this study may be due to variation in the sources of the lipoxygenases used in the separate experiment. Lipoxygenase used in this study was obtained from *Glycine max*, whereas that used in the previous report was obtained from potato tubers. With the lipoxygenase inhibitory effect of the *W. somnifera* root in this study supporting Khan et al. [51] previous investigations, the effect of compounds including sitoindoside IX in the plant with strong binding energy (−9.5 kcal/mol) for key amino acid residues of the inflammatory enzyme (Figure 8D), could be responsible for this observed enzyme inhibitory activity.

Diabetes is a metabolic disorder characterized by chronic hyperglycemia. Over a long time, diabetes has been documented to result in many complications that could affect many body organs. Neuropathy, as one of the long‐term complications of diabetes, has been linked with the development of Alzheimer's in patients suffering from the former metabolic disease [52]. It is therefore imperative that patients manage the underlying excessively high blood glucose level to lower the risk of neurodegenerative pathology. In this regard, drugs such as acarbose have been clinically approved to inhibit carbohydrate digestion and, consequently, the rate of glucose absorption into the blood. WSEE in Figure 5A showed potent inhibition of α‐glucosidase when compared with α‐amylase. When complex carbohydrates are broken down to oligosaccharides by α‐amylase, hydrolysis of the simple oligosaccharide molecules into monosaccharides capable of entering the blood is then carried out by α‐glucosidase. Although Oyewusi et al. [53], like in the present study, described the α‐glucosidase inhibitory effect of withanolide A, to the best of our knowledge, no previous investigation has explored the potential antidiabetic properties of *W. somnifera* withasomniferolide B, which showed binding affinity (−11.5 kcal/mol) stronger than acarbose (−7.3 kcal/mol) for the α‐amylase enzyme Table 2. Although lower binding energies may not directly correlate with greater inhibition, they could mediate closer interactions, increasing the likelihood of better inhibition between the ligand‐protein molecules than when they possess higher binding energies. As Lee et al. [54] reported, withasomniferol D, also isolated from *W. somnifera* root, showed anti‐adipogenic effects in 3T3‐L1 preadipocytes. These findings suggest that phytocannabinoids from the investigated plant could represent an alternative therapeutic means for managing hyperglycemia and obesity in type 2 diabetes.

A study by Onder et al. [55] confirmed the expression of the active BChE enzyme in neuroblastoma cell lines (SH‐SY5Y). Despite having lower substrate specificity for acetylcholine, as a key macromolecule that functions in chemical signaling between nervous and other cells, abnormally high levels of tumor‐related BChE have been described in children diagnosed with neuroblastoma [56]. When compared with BChE IC_50_ values between 29.0–85.2 µM reported for withanolide compounds isolated from *W. somnifera* plant [57], a similar estimate for the ethanol extract of the plant in this study was 313.6 μ/mL. Crude extract of any plant material contains a mixture of different classes of phytochemicals that could interfere with each other's biological activity. Thus, the higher inhibition reported in this previous finding is related to the better activity of the pure compounds compared to the crude extract tested in the present investigation. While suppressing BChE activity via showing increasing inhibition with increasing concentration (Figure 3), W*. somnifera* root extract further displayed significant reduction of SH‐SY5Y cells' viability to a level comparable with that of doxorubicin chemical standard, especially at its highest test concentration (Figure 6). In support of these current findings, Maarouf et al. [58] reported that a standardized root extract of the studied plant, when used as a vegetarian preparation, exhibited anticancer properties by activating a signaling pathway that led to apoptotic cell death in MDA‐MB231 and MCF‐7 breast cancer cells.

## 5 Conclusion

*Withinina somnifera* has been traditionally used as a medicinal plant for managing various ailments. The ethanol extract of the root in this study inhibits lipoxygenase, protein denaturation, and carbohydrate digestive enzymes. While the extract also showed cytotoxicity effects on SH‐SY5Y neuroblastoma cells, compounds such as somniferin and sitoindoside IX in the plant showed high affinities for the selected inflammatory and cholinergic target proteins. Despite the limitations of using crude extract and not characterizing the inherent phytochemicals in the studied plant, the outcomes of this study, which revealed strong interactions between withasomniferolide B and sitoindoside IX with respective α‐amylase and lipoxygenase in silico structures, need to be further explored using other experimental models. This latter scientific investigation could lead to the discovery of other new pharmacological uses for this herbal plant.

## Author Contributions

**Lufuno Mulelu**: investigation, data curation, methodology, and writing – original draft. **Jacqueline N. Manjia**: conceptualization, investigation, data curation, methodology, and writing – original draft. **Kolawole A. Olofinsan**: formal analysis, data curation, writing – original draft, software, and review and editing. **Londiwe Xulu**: formal analysis and methodology. **Motlalepula G. Matsabisa**: conceptualization, resources, supervision, funding acquisition, and review and editing.

## Patient Consent for Publication

No written consent has been obtained from patients as there are no patient identifiable data included in this article.

## Conflicts of Interest

The authors declare no conflicts of interest.

## Data Availability

The data that support the findings of this study are available from the corresponding author upon reasonable request.
